# 3D sheep rumen epithelial structures driven from single cells in vitro

**DOI:** 10.1186/s13567-023-01234-1

**Published:** 2023-11-09

**Authors:** Zebang Xu, Xinxin Xu, Bin Yang, Yuling Mi, Jiakun Wang

**Affiliations:** 1 Institute of Dairy Science, College of Animal Sciences, Zhejiang University, Hangzhou, 310058 China; 2 MoE Key Laboratory of Molecular Animal Nutrition, Zhejiang University, Hangzhou, China; 3 Department of Veterinary Medicine, College of Animal Sciences, Zhejiang University, Hangzhou, 310058 China; 4 School of Biological and Chemical Engineering, Zhejiang University of Science and Technology, Hangzhou, 310023 Zhejiang China

**Keywords:** Rumen epithelium, organoid, 3D culture, RNA-seq

## Abstract

Ruminants play a vital economic role as livestock, providing high-quality protein for humans. At present, 3D-cultured ruminant abomasum and intestinal organoids have been successfully established to study host and pathogen interaction. The rumen is a unique digestive organ of ruminants that occupies 70% of the volume of the digestive tract and its microbiota can decompose lignocellulose to support animal growth. Here we report a method for culturing rumen epithelial organoids. We found that single rumen epithelial cells form self-organized 3D structures representative of typical stratified squamous epithelium, which is similar to rumen epithelium. EGF, Noggin, Wnt3a, IGF-1, and FGF-10 significantly enhanced the seeding efficiency of organoids. Moreover, the inclusion of CHIR-99021, A83-01, SB202190, and Y-27632 is crucial for organoid formation and maintenance. Importantly, we demonstrate that rumen epithelial cells retain their ability to form organoids after passage, cryopreservation, and resuscitation. The rumen epithelial organoids express rumen cell type-specific genes, uptake fatty acids, and generate 2D cultures. In summary, our data demonstrate that it is feasible to establish organoids from single rumen epithelial cells, which is a novel in vitro system that may reduce the use of experimental animals.

**Supplementary Information:**

The online version contains supplementary material available at 10.1186/s13567-023-01234-1.

## Introduction

Organoids are considered to be complex self-organized 3D structures with organ-specific cell types and structural features that grow from stem cells in vitro [1]. Induction of intestinal organoids from Lgr5^+^ adult intestinal stem cells and recapitulation of cortical tissue development from pluripotent stem cells, stand as seminal accomplishments in the organoid field [2, 3]. With a better understanding of growth factor cocktails, extracellular matrix (ECM), and suspension culture, a large number of organoids from different cellular resources including esophageal organoids, gastric organoids, small intestinal organoids, colon organoids, liver organoids, kidney organoids, prostate organoids, brain organoids, optic cup organoids, thyroid organoids, and skin organoids, and different species including humans, mice, porcine, chicken, bovine, and ovine have been successfully established [4–7]. Organoids were chosen as the Method of the Year 2017 for their great prospects in development, disease modeling, drug screening, and regenerative medicine (2017) [8]. A fully defined, synthetic hydrogel based on a four-armed, maleimide-terminated poly macromer has been shown to support the growth and expansion of human intestinal organoids; this promising innovation presents a plausible avenue for diminishing reliance on tumor-derived Matrigel [9]. The development of organoids and synthetic hydrogels offers a good opportunity to effectively reduce the number of animals used for in vivo experiments, in line with the 3R principles.

Ruminant livestock possess an intricate and distinct gastric system comprising the rumen, reticulum, omasum, and abomasum. The rumen, reticulum, and omasum are identified as the forestomach to distinguish them from the monogastric system, and the microbiota inhabiting the forestomach can breakdown cellulose in plants and convert it into high-quality protein for human consumption. In contrast, the abomasum bears a resemblance to the stomach of monogastric animals. The use of stem cell-derived organoids as a new in vitro platform for ruminant research is an exciting development to investigate host-pathogen interactions [10]. A recent study reported the establishment of ovine abomasum organoids and demonstrated that the highly pathogenic ovine gastric parasite *Teladorsagia circumcincta* L3 specifically invades ovine abomasum organoids and resides in the lumen [11]. Faber and colleagues used bovine abomasum organoids to define the interactions between the abomasum epithelium and *Ostertagia ostertagi* L3 and demonstrated that *O. ostertagi* L3 expands the organoids prior to invasion through functionally active and heat-labile excretory-secretory products [12].

The rumen grows rapidly after birth and expands to occupy 70% of the stomach’s capacity, and the rumen microbiota provide ruminants with 70% of the daily energy required by fermentation in the form of short-chain fatty acids (SCFA) [13]. The rumen surface is covered with stratified squamous epithelium, comprising the stratum basale, stratum spinosum, stratum granulosum, and stratum corneum [14]. The keratinized surface can serve as a strong barrier against stimulation from roughage and microbiota [15]. Moreover, the rumen epithelium absorbs SCFA and converts them into ketone bodies as energy for animal growth [16]. Therefore, the rumen is an important target in ruminant research. However, no ruminant rumen organoid model has been previously reported.

In this study, we developed an in vitro culture system for the establishment of sheep rumen epithelial organoids from single cells. We demonstrate that organoids possess tissue-like epithelial architecture and cell types and reveal differences in transcriptional profiles between organoids and tissue by RNA-sequencing.

## Materials and methods

### Rumen epithelial cell isolation

Twelve lambs at 5, 10, 15, and 25 days of age (three lambs per age) were purchased from a commercial farm (Huzhou, Zhejiang, China). Lumianning (Hua Mu, Changchun, China) was injected intramuscularly at a dose of 2 µL/kg body weight. The lambs were bled to death after completely losing consciousness. Then the abdominal cavity was opened and the rumen was separated. After peeling off the serosa layer and muscle layer with forceps, approximately 1 g of rumen epithelial tissue was meticulously excised and promptly transferred into 2 mL polypropylene cryogenic vials (Corning, USA) and swiftly immersed in liquid nitrogen for subsequent RNA extraction.

Considering the higher cell isolation efficiency and lower risk of cell contamination, the rumen epithelial tissue from three lambs aged 5 days was selected as the object of rumen epithelial cell (REC) isolation. The rumen epithelial tissue was washed with 0.01 M sterile ice-cold phosphate-buffered saline (PBS; Biosharp, China) containing 1000 U/mL penicillin (Solarbio, China), 1 mg/mL streptomycin (Solarbio, China), 2.5 µg/mL amphotericin B (Yuanye Bio-Technology Co, China), and 500 µg/mL gentamycin (Yuanye Bio-Technology Co, China) until the solution appeared clean after washing. Then, the tissue was transported to the laboratory in modified Eagle medium (DMEM; Biosharp, China) containing 1000 U/mL penicillin, 1 mg/mL streptomycin, 2.5 µg/mL amphotericin B, and 500 µg/mL gentamycin.

The rumen epithelial tissue was cut into small pieces (5 mm × 5 mm), washed with ice-cold PBS five times, transferred to 0.25% trypsin-EDTA solution (Solarbio, China) containing 10 µΜ Y-27632 (MCE, USA) and shaken in a water bath at 37 °C. The digested solution was collected and replaced with fresh solution every 20 min to eliminate dead cells and keep only alive dissociated cells with a regular, round aspect. Trypsinization was terminated after collecting the digested solution 8–10 times. The collected solution was filtered with a 70-µm cell strainer (Biosharp, China) and centrifuged at 300 × *g* for 5 min at 4 °C. The supernatant was discarded, and the pellet was resuspended in epithelial cell culture medium (ECCM: DMEM supplemented with 10% (v/v) fetal bovine serum (FBS; Genom, China), 5 µg/mL insulin (human; MCE), 10 ng/mL EGF (human; MCE), 10 µM Y-27632, 100 U/mL penicillin, and 0.1 mg/mL streptomycin). The cells were seeded at a density of 5 × 10^4^ cells/mL on 100 mm TC-treated culture dishes (Corning, USA). The culture dishes were incubated at 37 °C and 5% CO_2_ and the ECCM was changed every 2 or 3 days.

### Cell resuscitation, passage and cryopreservation

For resuscitation, the 2 mL polypropylene cryogenic vials stored in liquid nitrogen were removed, and the cell cryopreservation medium (consisted of 90% (v/v) FBS and 10% (v/v) dimethylsulfoxide (Solarbio, China) containing 10 µM Y-27632) was mixed with DMEM after thawing by rapid agitation in a 37 °C water bath and then centrifuged at 300 × *g* for 5 min at 4 °C. The supernatant was discarded, and the pellet was resuspended in ECCM and seeded at a density of 5 × 10^4^ cells/mL on 100 mm TC-treated culture dishes. For passage, the medium was discarded, and the dish was washed twice with PBS. TrypLE™ Express (Gibco; USA) was added to the dish and incubated at 37 °C for 10 min. Then, the TrypLE™ Express was transferred to a 15 mL centrifuge tube (Corning, USA) and centrifuged at 300 × *g* for 5 min at 4 °C. The pellet was resuspended in ECCM and seeded at a density of 5 × 10^4^ cells/mL on 100 mm TC-treated culture dishes. For cryopreservation, the digested cells were suspended in freshly prepared cell cryopreservation medium at a density of 1 × 10^6^ cells/mL and transferred to cryogenic vials, which were placed in a programmed cooling box at -80 °C overnight and stored in liquid nitrogen.

### Organoid culture and passaging

The cells cultured on the dishes were digested and resuspended in organoid culture medium (OCM: Advanced DMEM/F-12 (Gibco, USA) supplemented with 1 × N2 (Gibco, USA), 1 × B27 (Gibco, USA), 1 × GlutaMax (Gibco, USA), 100 U/mL penicillin, 0.1 mg/mL streptomycin, 0.01 M HEPES (Solarbio, China), 10 mM nicotinamide (Sigma, USA), 1 mM N-acetyl-L-cysteine (Sigma, USA), 50 ng/mL EGF, 100 ng/mL Noggin (human; MCE), 100 ng/mL Wnt3a (human; MCE), 100 ng/mL R-spondin1 (human; MCE), 100 ng/mL IGF-1 (human; MCE), 100 ng/mL FGF-10 (human; MCE), 3 µM CHIR-99021 (MCE), 5 µM A83-01 (MCE), 10 µM SB202190 (MCE) and 10 µM Y-27632) at a density of 2 × 10^5^ cells/mL. The cell suspension was mixed with Growth Factor Reduced Matrigel (Corning, UK) on ice at a ratio of 1:4 (v/v) and placed as a 50 µL droplet in a 24-well TC-treated multiple well plate (Corning, USA) followed by incubation at 37 °C for 30 min to ensure solidification of the gel. Then, 500 µL of OCM was added to each well and incubated at 37 °C and 5% CO_2_. The OCM was changed every 2 or 3 days during the experimental period. To passage rumen epithelial organoids, the OCM was removed and 1 mL of cell recovery solution (Corning, USA) was added to each well to depolymerise the Matrigel. The resulting suspension containing the organoids was collected into a 15 mL centrifuge tube and centrifuged at 300 × *g* for 3 min at 4 °C. The pelleted organoids were resuspended in TrypLE™ Express and dissociated into single cells after a 30 min incubation at 37 °C, and the single cells were seeded as described above. Images were acquired with a microscope (TE2000-U, Nikon, Japan) every day. The organoid formation rate was calculated as the percentage of the organoid number over the number of cells seeded. The organoid diameter was measured with Image-Pro Plus software.

### Generation of 2D organoid cultures

After 9 days of culture, organoids were collected from Matrigel with a cell recovery solution. Organoids were resuspended in OCM at a density of 3000 organoids/mL, 200 µL of organoid suspension was added to the Matrigel coated 24-well cell culture inserts (Corning, USA) and 700 µL of OCM was added to the cell culture insert companion plates (Corning, USA). The cell culture insert coating procedure involved incubation of 2% Growth Factor Reduced Matrigel (v/v) in advanced DMEM/F-12 at 37 °C and 5% CO_2_ for 1 h after which the liquid was removed. The OCM was changed every day, and the polyethylene terephthalate (PET) membranes of the cell culture inserts were cut with a scalpel on d7 for immunofluorescence staining.

### Fatty acid absorption assays

To demonstrate the absorptive capacity, the 3D organoids and 2D cultures were incubated for 30 min with the BODIPY 500/510 C1, C12 probe (MCE, USA) to a final concentration of 10 µM, 0.1% DMSO was used as a control treatment. Then the organoids and 2D cultures were rinsed with PBS for 5 min 3 times. The images were collected using a fluorescence microscope (IX70, Olympus, Japan).

### Sample preparation for histology

The collected organoids, tissue, and PET membranes were fixed with 4% paraformaldehyde (Beyotime, China) for 1 day at 4 °C. Then, they were washed with PBS and dehydrated in 15% sucrose for 2 d and 30% sucrose for 3 days at 4 °C. Then, the samples were embedded in optimal cutting temperature compound (OCT; SAKURA, USA) and stored at − 80 °C. Frozen blocks were sectioned into 10 μm thick sections using a cryostat (Thermo Fisher Scientific, NX50) and attached to adhesion microscope slides (CITOTEST, China).

### Immunofluorescence staining assay

Immunofluorescence staining of cells cultured in 96-well plates: The cells were fixed with 4% paraformaldehyde for 15 min, washed with PBS for 5 min 3 times, permeabilized with 0.5% Triton X-100 for 20 min and washed with PBS for 5 min 3 times. After blocking with 100 µL goat serum (Beyotime, China) for 30 min, the cells were incubated with the primary antibody (diluted in PBS) at 4 °C overnight. The next day, the primary antibody was removed, and the cells were washed with PBS for 5 min 3 times. Then, the cells were incubated with secondary antibody (diluted in PBS) at 37 °C for 1 h and washed 3 times with PBS for 5 min each time. Finally, the cells were stained with DAPI for 20 min and washed with PBS for 5 min 3 times. Images were acquired with a microscope (IX70, Olympus, Japan).

Sections were subjected to antigen retrieval with 0.01 M citrate buffer (pH = 6.0) at 99 °C for 5 min and washed with PBS-Triton-X for 15 min. The sections were then blocked with PBB (containing PBS, 0.05% Triton X-100 and 0.5% BSA)-5% goat serum for 45 min. After incubation with the primary antibody (diluted in PBB) at 4 °C overnight, the sections were washed with PBS for 30 min and then incubated with secondary antibody and DAPI (diluted in PBB) for 1 h. The sections were then washed with PBS for 10 min 3 times and mounted with Antifade Mounting Media (Beyotime, China), and images were acquired with a microscope (80i, Nikon, Japan).

Cells, organoids, and tissues treated only with the isotype controls (rabbit IgG) were negative of any labelling (Additional file 1). The antibodies used for immunofluorescence staining are shown in Table 1.

**Table 1:** **List of antibodies used for immunofluorescence staining**

Antibodies	Source	Identifier	Dilution
KRT14 Rabbit pAb	ABclonal	A15069	1:200
IVL Rabbit pAb	ABclonal	A8026	1:200
ZO-1 Rabbit pAb	ABclonal	A0659	1:200
Ki67 Rabbit pAb	ABclonal	A11390	1:100
Cy3 Goat Anti-Rabbit IgG (H + L)	ABclonal	AS007	1:200
DAPI	Beyotime	C1002	5 µg/mL
Rabbit IgG	Beyotime	A7016	1:100

### Transmission electron microscopy (TEM)

The sample was fixed with 2.5% glutaraldehyde overnight, washed in PBS for 15 min three times, postfixed with 1% OsO4 for 1 h and again washed in PBS for 15 min three times. The sample was first dehydrated by a graded series of ethanol (30%, 50%, 70%, 80%) for approximately 15 min at each step and then dehydrated by a graded series of acetone (90%, 95%) for approximately 15 min at each step. Finally, the samples were dehydrated twice with absolute acetone for 20 min each time. The sample was placed in a 1:1 mixture of absolute acetone and the final Spurr resin mixture for 1 h and then transferred to a 1:3 mixture of absolute acetone and the final resin mixture for 3 h and to the final Spurr resin mixture overnight. The sample was sectioned in a LEICA EM UC7 ultratome, and the sections were stained with uranyl acetate and alkaline lead citrate for 10 min and observed with a Hitachi Model H-7650 TEM.

### mRNA extraction and transcriptome analysis

P1 organoids from three lambs aged 5 d were recovered from Matrigel, resuspended in TRIzol (Aidlab, Beijing, China) and stored in liquid nitrogen for RNA extraction. Total RNA of organoids and tissue was extracted using a total RNA extraction kit (Aidlab, Beijing, China). The RNA quality was evaluated by an Agilent 2100 bioanalyzer (Agilent Technologies, CA, USA). RNA sequencing was performed using the Illumina platform by Novogene (Beijing, China), and clean reads were obtained by removing low-quality reads (base quality < Q20 bases), adapters, and reads with more than 10% unknown nucleotides (N). The rumen epithelial tissue transcriptome data of 5, 10, 15, and 25-day-old lambs (*n* = 3) were available in another study in our laboratory [17]. Salmon v.1.5.1 [18] was used to align the clean reads to the sheep genome (Oar_rambouillet_v1.0) to determine transcripts per million (TPM) in individual biological samples. Genes that appeared in less than half of the samples were filtered.

Differentially expressed genes (DEG) between organoids and tissue were determined using DESeq2 v.1.36.0 [19] with |log_2_fold change| > 1 and false discovery rate (*FDR*) < 0.05. The tissue was grouped for the different ages and compared with organoids respectively and then 4 sets of DEG were obtained, the union of the 4 sets of DEG was used for weighted gene coexpression network analysis (WGCNA) in R [20]. To generate coexpression modules, the settings were as follows: minModuleSize = 30, merCutHeight = 0.2 and deepSplit = 1. The genes in the modules were enriched based on the Kyoto Encyclopedia of Genes and Genomes (KEGG) using the Database for Annotation, Visualization, and Integrated Discovery (DAVID). The KEGG pathways with *P* < 0.05 were considered significantly enriched, and the top 20 KEGG pathways were collected. The results were visualized by the OmicStudio Cloud Platform [21].

### Statistical analysis

The data are presented as the mean ± SD. Data on organoid formation rates and diameters were assessed to be normally distributed by the Shapiro-Wilk test and then were subjected to multiple comparisons using the Tukey HSD test by IBM SPSS statistics 25 software (IBM Corp, NY, USA). Significance was defined as *P* < 0.05.

## Results

### In vitro cultivation of rumen epithelial organoids

**Figure 1:**
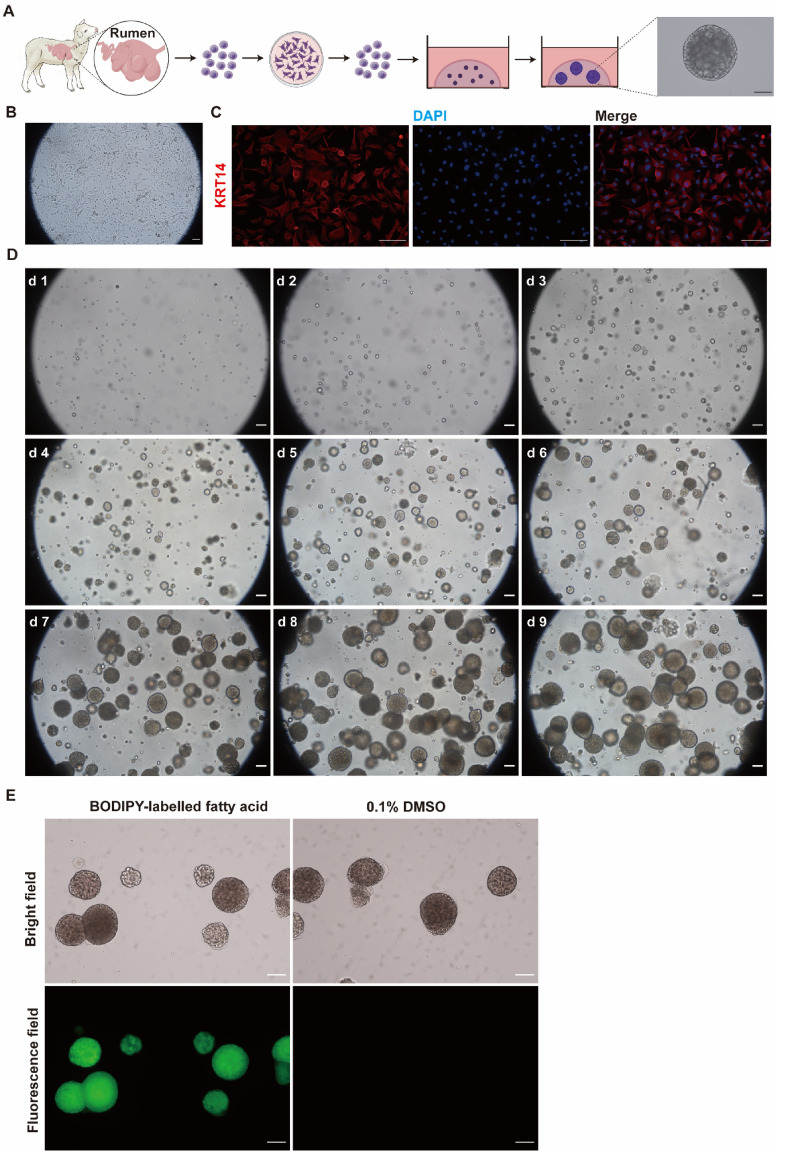
** In vitro growth of rumen organoids. A** Experimental schematic of the development of rumen organoids from single cells (Drawn by Figdraw). **B** Bright field of rumen epithelial cells cultured on dishes. **C** Immunostaining of keratin 14 (KRT14) and DAPI for rumen epithelial cells. **D** Representative images showing organoid growth and development. **E** Fatty acid uptake of organoids. Scale bars = 100 μm. Representative images from three independent experiments using three individual lambs.

The REC were isolated from the rumen tissue of 5-day-old lambs and embedded in Matrigel and cultivated into organoids (Figure 1A). Freshly isolated REC were first cultured on cell culture dishes to remove dead cells and fibroblasts. After 24 h of culture, surviving REC began to proliferate and generate clusters (Figure 1B), and typical characteristics of cobblestone morphology were observed during continued culture. The REC exhibited strong immunopositive staining for keratin 14 (KRT14), which is a marker of rumen epithelial basal cells (Figure 1C). The expression of Involucrin (IVL) in REC was not observed by fluorescent staining (Additional file 1). Within 2 days of culture, single REC began to proliferate into small cell masses. After 9 days of continuous culture, single cells formed spherical structures with a diameter of more than 200 μm (Figures 1A, D). Fluorescent labels within the organoids were visualized after incubation with BODIPY-labelled fatty acid probe (Figure 1E).

**Figure 2:**
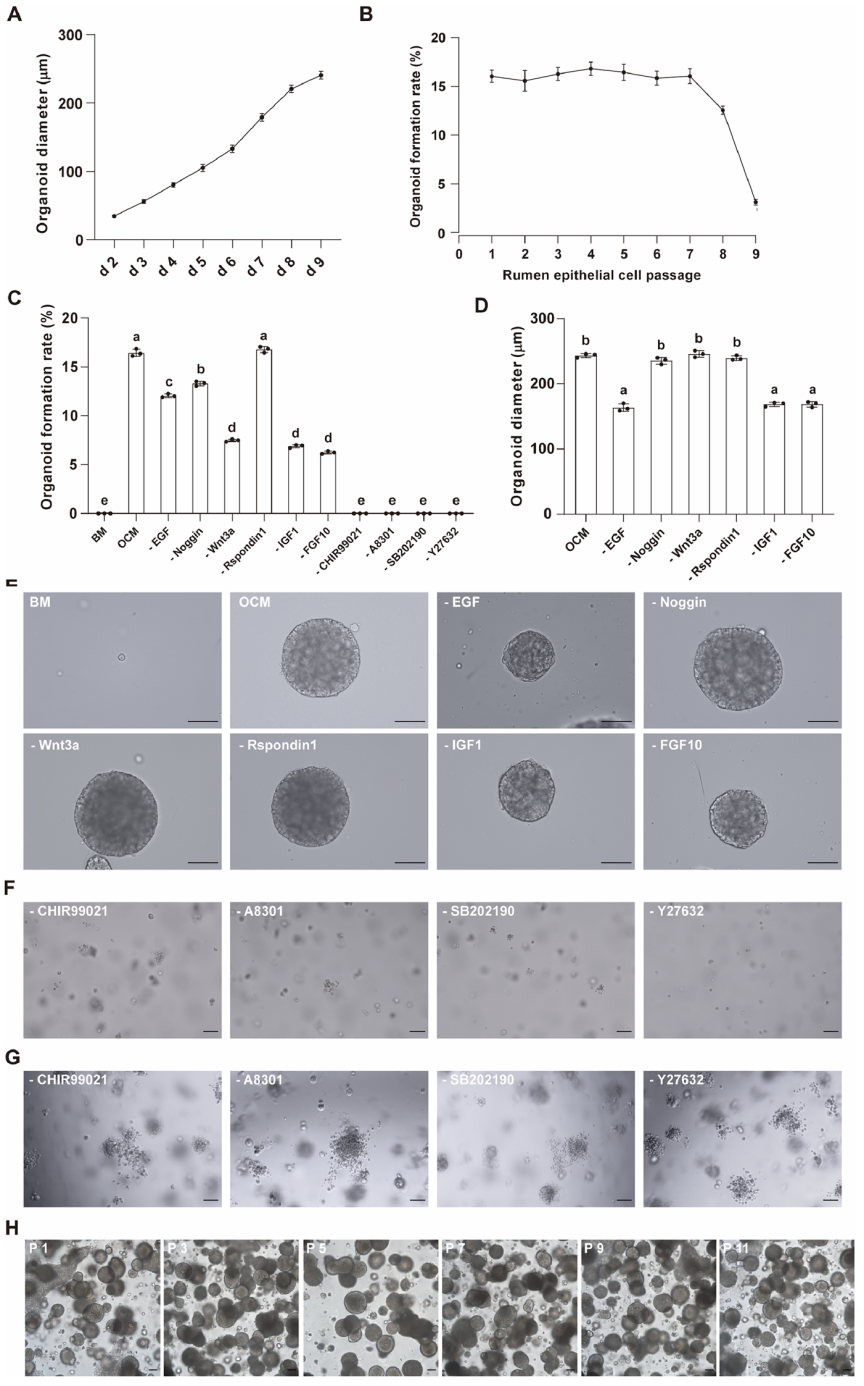
** Rumen organoids cultured in minus medium. A** Diameter variation of organoids. **B** Organoid formation rates of rumen epithelial cells after multiple passages. **C, D** Formation rates and diameters of organoids cultured in minus medium (different superscript letters indicate significant differences, *P* < 0.05). **E, F** Organoids cultured in minus medium. **G** Inhibitor removal from OCM for 3 days. **H** Representative images showing organoids following ten passages. Scale bars = 100 μm. Data and images are representative of three independent experiments using three individual lambs.

The REC that had been cryopreserved, resuscitated and passaged, preserved the potential to generate organoids, and the organoid formation rates of REC from the passages 1–7 were above 15% (Figure 2B). After 9 passages, REC began to age and gradually lost the ability to sustain expansion, and the proportion of cells that could form organoids also dropped to less than 5%. To analyze the importance of a panel of growth factors and inhibitors added to the OCM, we then withdrew each of the components from the OCM. We found that compared with REC cultured in OCM, REC cultured in basic medium (BM, Table 2) were unable to form organoids, and the rate of organoid formation decreased significantly after removing EGF, Noggin, Wnt3a, IGF-1 or FGF-10 (*P* < 0.05), while removing R-spondin1 did not affect the organoid formation rate (*P* > 0.05, Figure 2C). The organoid size was significantly reduced after removing EGF, IGF-1 or FGF-10 (*P* < 0.05), while removing Noggin, Wnt3a or R-spondin1 had no significant effect on the organoid size (*P* > 0.05, Figures 2D and E). Organoids failed to form after removing the inhibitors CHIR-99021, A83-01, SB202190 or Y-27632, and some REC disintegrated in the absence of any inhibitor (Figures 2C, F). In addition, we removed CHIR-99021, A83-01, SB202190 or Y-27632 from the medium after culturing organoids with OCM for 9 days, and the disintegration of all organoids was observed within 3 days (Figure 2G). Furthermore, we verified the feasibility of organoid passage. After 10 passages, organoids retained the original morphological characteristics (Figure 2H).

**Table 2:** **List of reagents to prepare BM and OCM**

BM	Final	OCM (BM +)	Final
Advanced DMEM/F-12		EGF	50 ng/mL
Penicillin/streptomycin	1 ×	Noggin	100 ng/mL
N2	1 ×	Wnt3a	100 ng/mL
B27	1 ×	R-spondin1	100 ng/mL
GlutaMax	1 ×	IGF-1	100 ng/mL
HEPES	1 ×	FGF-10	100 ng/mL
Nicotinamide	10 mM	CHIR-99021	3 µM
N-Acetyl-L-Cysteine	1 mM	A83-01	5 µM
		SB202190	10 µM
		Y-27632	10 µM

### Morphology of tissue and organoids

**Figure 3:**
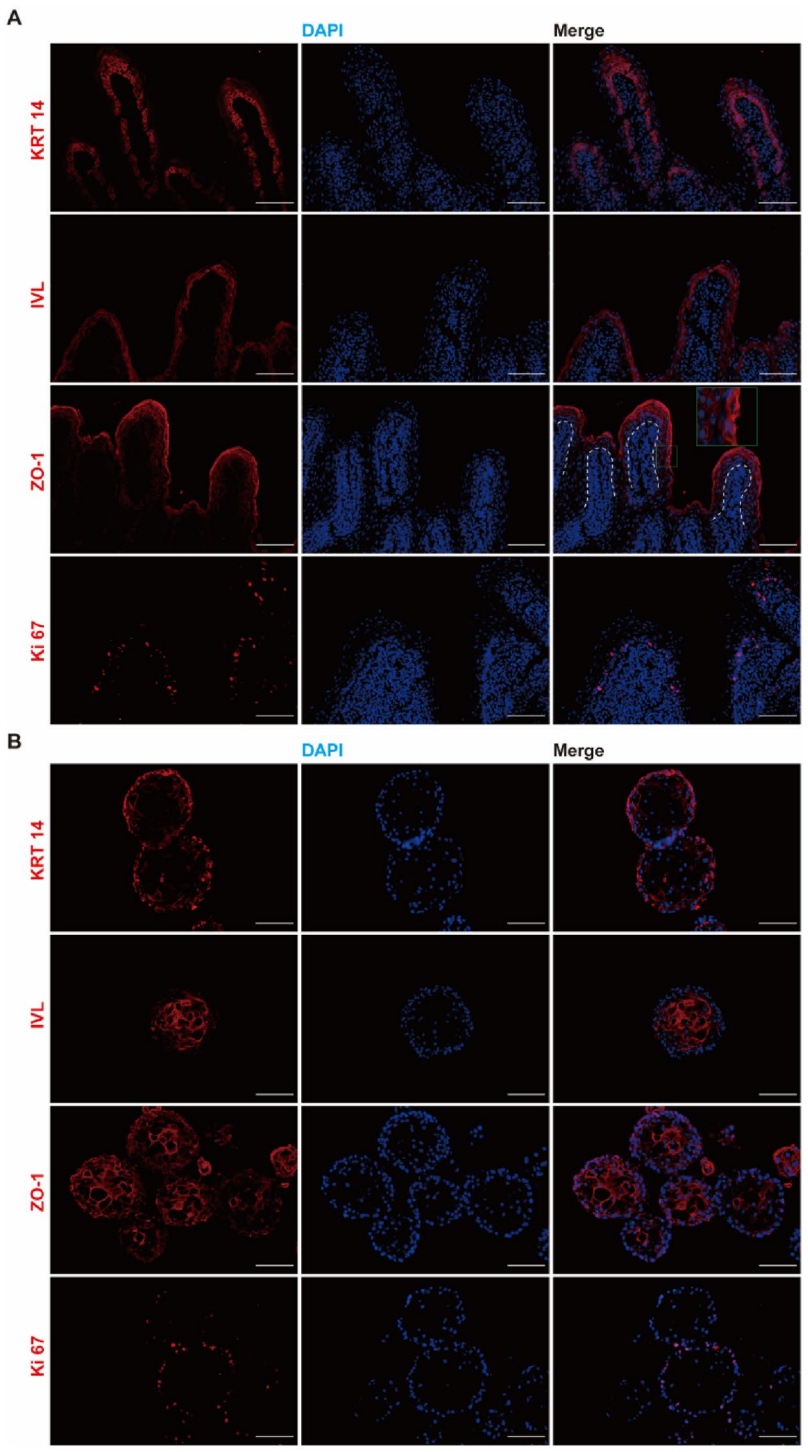
** Characterization of rumen epithelial tissue and rumen organoids by immunofluorescence. A** Rumen epithelial tissue. **B** Rumen organoids. Scale bars = 100 μm. Representative images from three independent experiments using three individual lambs.

Immunofluorescence staining of rumen epithelial tissue and rumen epithelial organoids is shown in Figure 3. The stratum basale consisted of a monolayer of cells and had positive fluorescence for KRT14 (Figure 3A). Cells above the stratum basale were marked by Involucrin (Figure 3A). The major components of tight junction zonula occludens-1 (ZO-1) were detected, and the fluorescence intensity of the stratum corneum was stronger than that of the stratum basale (Figure 3A). The proliferation marker Ki67 was detected in a few cells of the stratum corneum, indicating that the proliferation of rumen epithelial cells occurs in the stratum corneum (Figure 3A). Organoids were spherical structures consisting of multiple layers of cells. The outermost cells were marked by KRT14, and the inner cells were marked by IVL (Figure 3B). As in tissue, ZO-1 was detected in organoids, indicating tight junctions between cells (Figure 3B). Ki67 was detected in the outermost cells, indicating that the cell division of organoids occurs in the outer layer (Figure 3B).

**Figure 4:**
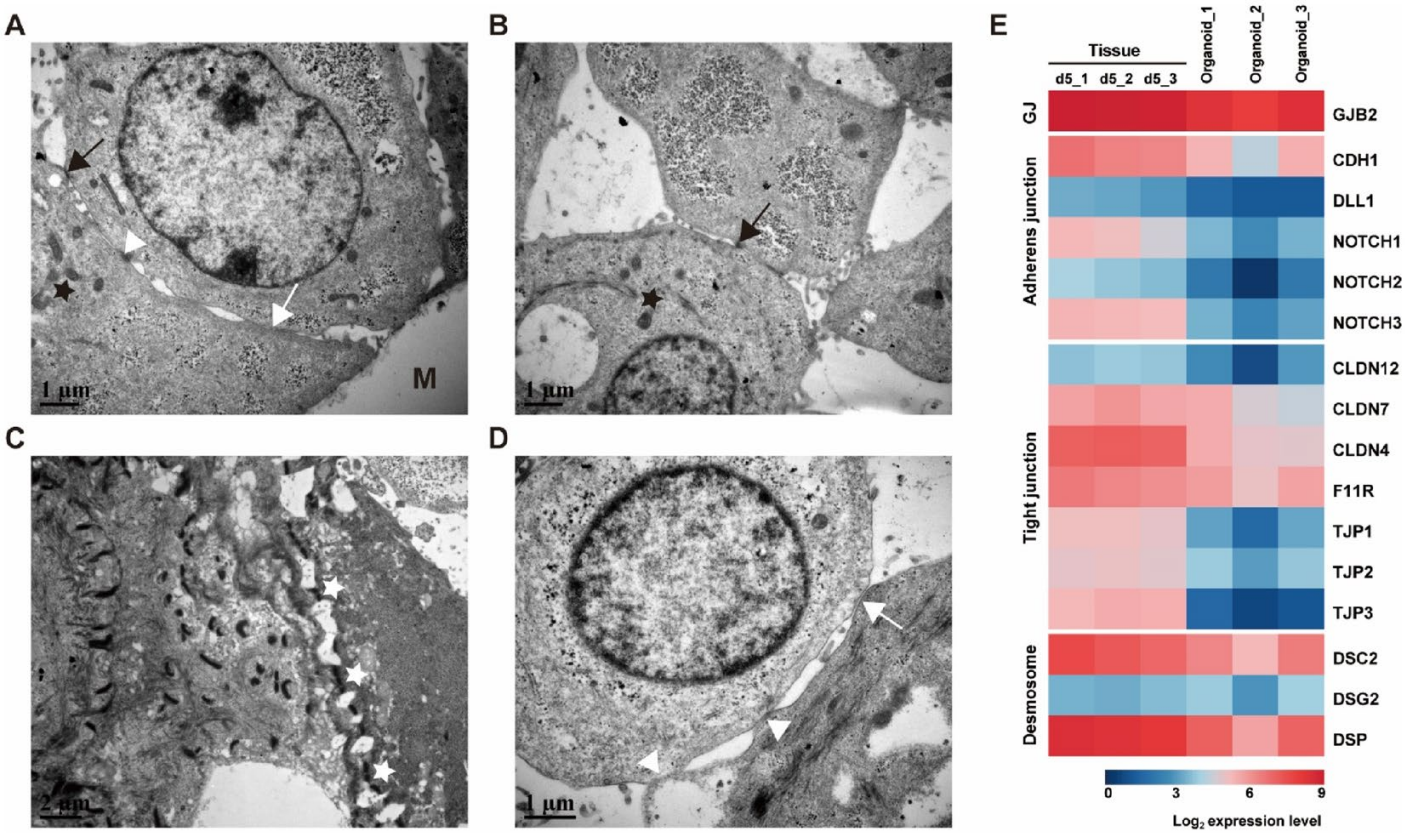
**Ultrastructural analysis and expression of mRNA encoding cell junction-related genes in rumen epithelial tissue and organoids.** **A** Transmission electron microscopy (TEM) showing the tight junctions, desmosomes, and gap junctions between cells in the outer layer of the organoid and the mitochondria in the cytoplasm. **B** TEM showing the tight junctions between cells and the mitochondria inside the organoid, with gaps of a few micrometers between cells. **C** TEM showing the dense corneodesmosomes between cells in the inner layer of the organoid. **D** TEM showing the tight junctions and gap junctions between cells inside organoids. **E** Heatmap showing the relative expression levels (log_2_ transcripts/million reads) of a range of epithelial cell junction-related genes. GJ, gap junction. M, Matrigel; Black arrow, desmosome; White arrow, gap junction; Black star, mitochondria; White star, corneodesmosomes; White triangle, tight junction. Representative TEM images of one experiment. The expression data include rumen tissue and rumen organoids from 3 lambs at 5 days of age.

Transmission electron microscopy (TEM) revealed the ultrastructure of the organoids. The cells in the outer layer of the organoids were connected by desmosomes (black arrows), tight junctions (white triangles), and gap junctions (white arrows), and mitochondria were abundant in the cytoplasm (Figure 4A). In the inner layer of the organoids, tight junctions, gap junctions and desmosomes were detected between cells, while dense corneodesmosomes (white star) existed between cells (Figures 4B, C, and D). In addition, there were gaps of a few micrometers between cells in the inner layer of the organoids (Figures 4B, D).

We next compared gene expression related to cell junctions in rumen organoids and rumen tissue (from lambs aged 5 days) by mRNA sequencing (Figure 4E). Expression of genes encoding gap junctions, adherens junctions, tight junctions and desmosome components was detected in both organoids and tissue, consistent with the ultrastructure detected by TEM (Figures 4A, E).

### Generation of rumen epithelial 3D-derived 2D organoid cultures

To establish the 2D organoid culture system, 3D organoids were seeded on the Matrigel coated cell culture inserts and OCM was added to maintain the 2D cultures (Additional file 2). After 5–7 days of culture, confluent cell layers spread from the organoids seeded on the cell culture insert were observed which could be maintained for a further 7 days. Disruption of the 2D cultures was observed when the culture time exceeded 20 days. The stratified KRT14^+^ cells and IVL^+^ cells were observed by immunofluorescent staining of longitudinal sections of 2D cultures. The staining results for ZO-1 and Ki67 indicated the presence of tight junction formation and cell proliferation in 2D cultures. 2D cultures retain the same ability to uptake fatty acids as 3D organoids.

### Expression of rumen epithelial cell subpopulation specific genes in organoids and tissue

Single-cell sequencing has been used to characterize markers of different cell populations in tissue and organs; therefore, we used the sheep rumen epithelial lineage-specific gene sets reported by Yuan et al. [22] to help gain a better understanding of the cellular components of rumen epithelial organoids.

**Figure 5:**
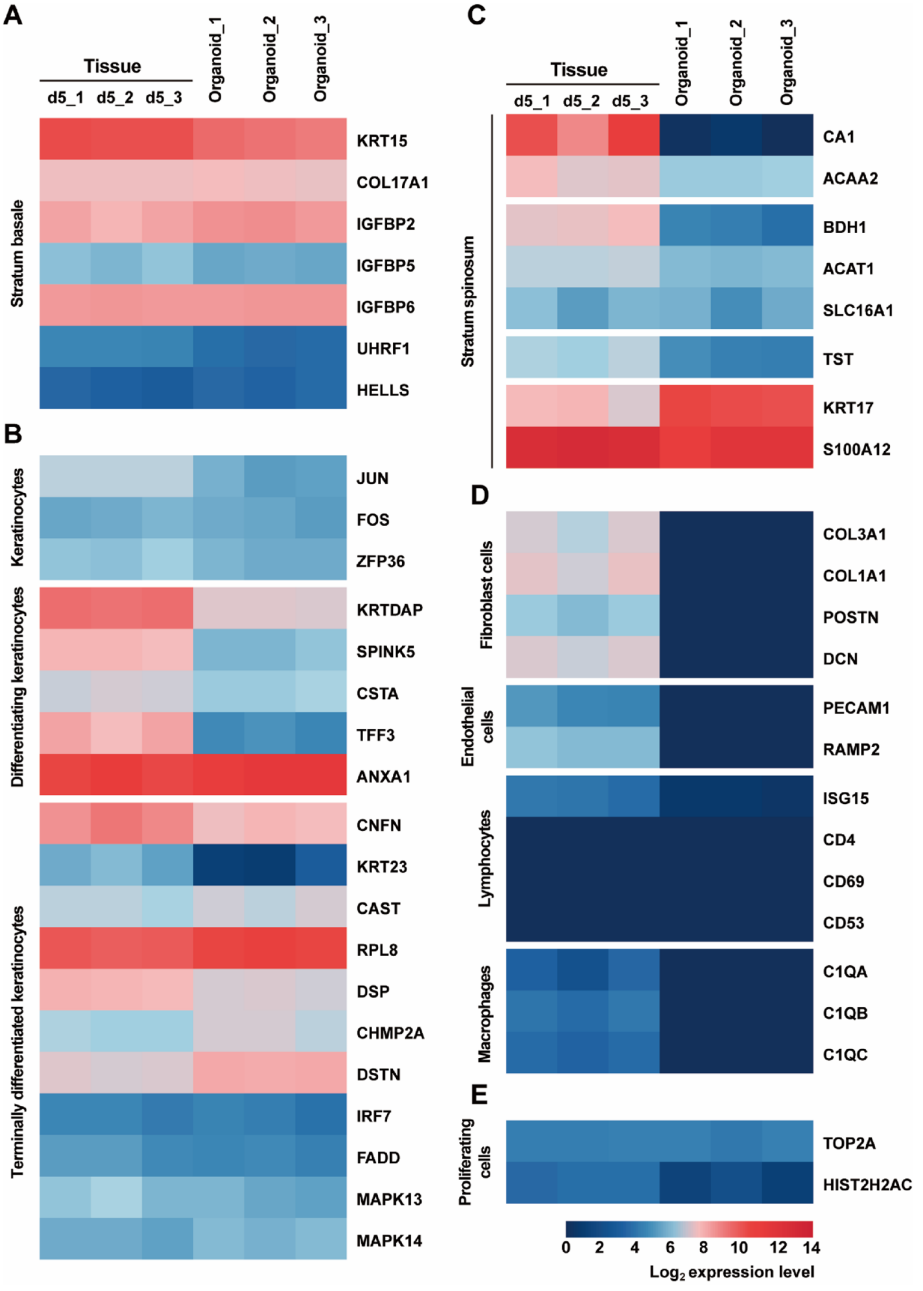
** Expression of rumen epithelial cell lineage-specific genes in tissue and organoids. A** Heatmap showing the relative expression levels (log_2_ transcripts/million reads) of a range of rumen epithelial cell lineage-specific genes of stratum basale, **B** stratum granulosum & corneum, **C** stratum spinosum, **D** non-epithelial cells, **E** proliferating cells. The expression data include rumen tissue and rumen organoids from 3 lambs at 5 days of age.

The stratum basale is a single layer of columnar cells adjacent to the basal lamina [14]. We found that the expression levels of basal cell-specific genes, including *KRT15*, *COL17A1*, *IGFBP2*, *IGFBP5*, *IGFBP6*, *UHRF1*, and *HELLS*, were similar in organoids and tissue, indicating the presence of basal cells in organoids (Figure 5A), which is consistent with the immunofluorescence results (Figure 3).

Keratinocytes proliferate and differentiate from the stratum basale and migrate to the upper layer, and their terminally differentiated products form the stratum corneum [23]. Similar expression levels of the keratinocyte-related genes *JUN*, *FOS*, and *ZFP36* were detected in tissue and organoids, implying the presence of keratinocytes in the organoids (Figure 5B). The expression of specific genes associated with differentiated keratinocytes (*KRTDAP*, *SPINK5*, *CSTA*, *TFF3*, and *ANXA1*) and terminally differentiated keratinocytes (*CNFN*, *KRT23*, *CAST*, *RPL8*, *DSP*, *DSTN*, *IRF7*, *FADD*, *MAPK13*, and *MAPK14*) was detected in both organoids and tissue, suggesting that corneocytes may be present in organoids (Figure 5B), consistent with the results shown in Figure 3.

The stratum spinosum is the main metabolic layer of the rumen epithelium. We analyzed the expression of specific genes related to the cells of the stratum spinosum and found that the expression levels of genes related to metabolism, including *CA1*, *ACAA2*, *BDH1*, *ACAT1*, *SLC16A1*, and *TST*, gradually increased with age in tissue, while the expression levels of those genes in organoids were relatively low (Figure 5C). In contrast, abundant expression of the cornification-related genes *KRT17* and *S100A12* was detected in tissue and organoids (Figure 5C).

Furthermore, the expression of specific genes related to fibroblasts (*COL3A1*, *COL1A1*, *POSTN*, and *DCN*), endothelial cells (*PECAM1* and *RAMP2*), lymphocytes (*ISG15* and *CD53*), and macrophages (*C1QA*, *C1QB*, and *C1QC*) was detected in tissue but absent in organoids (Figure 5D). The lymphocyte-related genes *CD4* and *CD69* were absent in both tissue and organoids (Figure 5D).

The expression of the cell cycle-related genes *TOP2A* and *HIST2H2AC* was detected in tissue and organoids, suggesting the presence of proliferating cells in tissue and organoids (Figure 5E), which was verified by immunofluorescence (Figure 3).

### Differential expression of mRNA in tissue and organoids revealed by WGCNA

**Figure 6:**
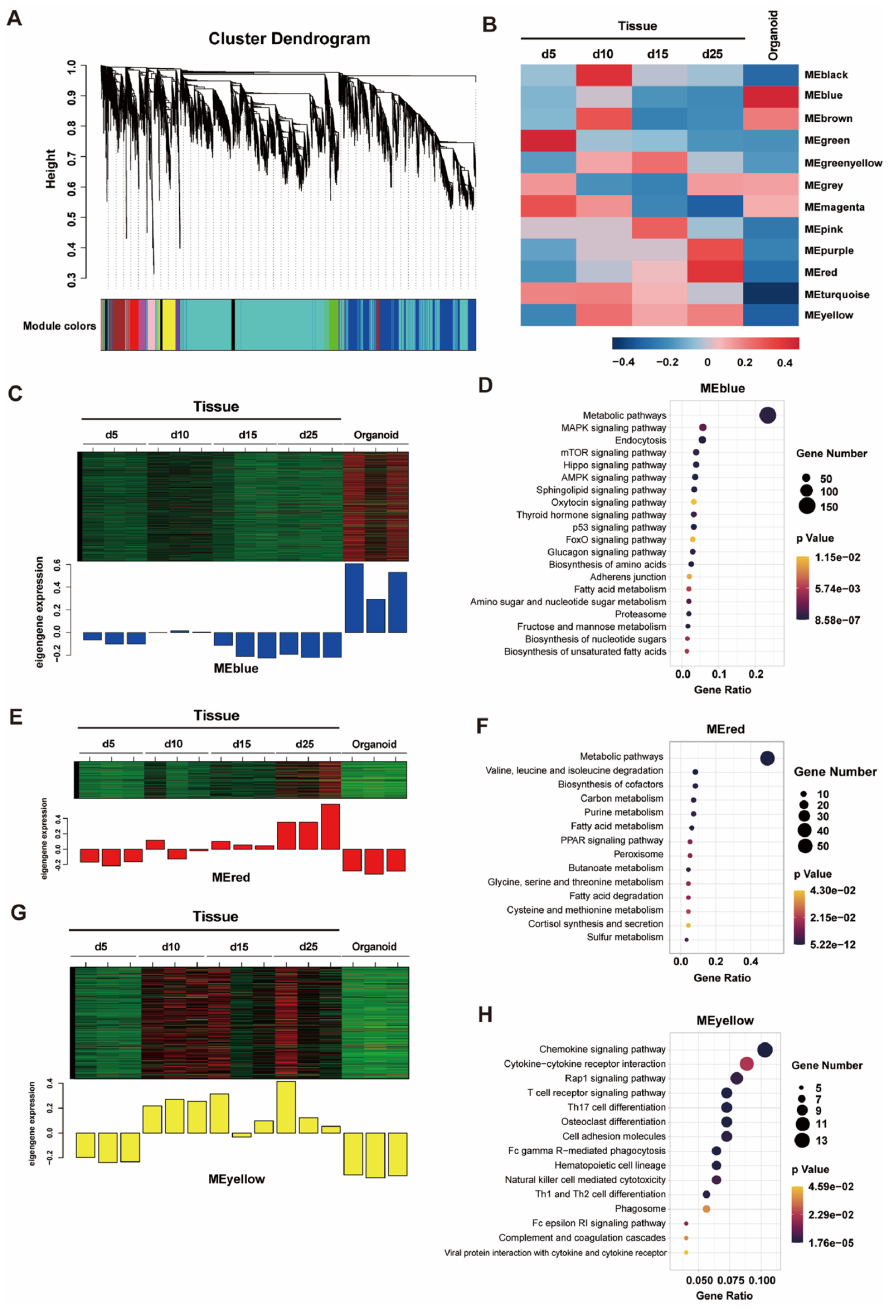
** WGCNA of rumen epithelial tissue and organoids. A** Clustering dendrogram of genes, with dissimilarity based on topological overlap, together with assigned module colors. **B** Heatmap showing the eigengene expression levels of 12 modules in 5 groups. **C** Eigengene expression level of MEblue in 15 samples. **D** KEGG pathway analysis of MEblue. **E** Eigengene expression level of MEred in 15 samples. **F** KEGG pathway analysis of MEred. **G** Eigengene expression level of MEyellow in 15 samples. **H** KEGG pathway analysis of MEyellow. RNA sequencing data contains rumen tissue from lambs at 5, 10, 15, 25 days of age and organoids from lambs at 5 days of age (*n* = 3).

To confirm transcriptional profile differences between organoids and tissue of different ages, WGCNA was used for DEG clustering. A total of 12 modules composed of highly related genes were identified by WGCNA (Figure 6A). Each module was summarized by an eigengene, which was the first principal component in the module [20]. MEblue has the largest difference between tissue and organoids, MEred and MEyellow are similar between 5 days tissue and organoids while have large differences from 10, 15, and 25 days tissue. The expression of the eigengene is shown in Figure 6B. Genes in MEblue were highly expressed in organoids and expressed at low levels in tissue (Figure 6C). Metabolism-related pathways, including biosynthesis of unsaturated fatty acids, biosynthesis of nucleotide sugars, fructose and mannose metabolism, amino sugar and nucleotide sugar metabolism, biosynthesis of amino acids, and fatty acid metabolism, and signal transduction-related pathways, including the FoxO signaling pathway, sphingolipid signaling pathway, AMPK signaling pathway, Hippo signaling pathway, mTOR signaling pathway, and MAPK signaling pathway, were enriched (Figure 6D). Gene expression levels in MEred and MEyellow were increased with age in tissue, and there were similar expression levels in 5-day-old lamb tissues and organoids (Figures 6E, G). The enriched pathways in MEred were mainly related to amino acid, carbon, purine, and fatty acid metabolism (Figure 6F). In addition, the PPAR signaling pathway and cortisol synthesis and secretion, which mainly regulate glucose and lipid metabolism, were also enriched (Figure 6F). The KEGG pathways enriched in MEyellow, such as the chemokine signaling pathway, T-cell receptor signaling pathway, and Th17 cell differentiation, were mainly related to the immune system (Figure 6H).

## Discussion

Ruminants are an important source of animal-source food that has the ability to produce high-quality protein that is of high value to humans from feed that is of little value to humans [24]. As an important digestive organ of ruminants, the rumen is the main site of energy metabolism and an important target of ruminant nutrition research. In this study, we demonstrate the ability to generate organoids from a single REC. To our knowledge, this is the first demonstration of organoids representing the rumen of a ruminant.

The rumen epithelium is a stratified squamous epithelium, and keratinocytes originating from the stratum basale have the potential to proliferate and differentiate, which makes it possible to isolate cells from the stratum basale for 3D culture [25]. The REC that have been passaged, cryopreserved, and resuscitated maintain the ability to generate organoids in vitro, organoids can be serially passaged more than 10 times and maintained in culture for several months, which means that cells isolated from several lambs can satisfy a large number of subsequent experiments, greatly reducing the use of experimental animals.

Epidermal growth factor (EGF), the proto-oncogene Wnt3a, the Wnt signal amplifier R-spondin1, and the bone morphogenetic protein inhibitor Noggin are the basic components of organoid cultivation [26]. Wnt3a and R-spondin1 can stimulate intestinal crypt cell proliferation and maintain the stem cell state [27], Noggin can stimulate intestinal crypt formation [28], and EGF can stimulate intestinal epithelial cell proliferation and block stem cell apoptosis [29]. A lack of EGF, Noggin, R-spondin1, or Wnt3a in human gastric organoid culture strongly reduces the efficiency of organoid formation [30]. Similar results were observed in our study. A lack of EGF, Noggin, or Wnt3a significantly reduced the efficiency of rumen epithelial organoid formation, and sustained EGF loss significantly affecting the growth of organoids. In contrast, the loss of R-spondin1 had no effect on the growth of organoids, which may be due to differences in the species and organ sources used in our study compared to those of prior studies. Insulin-like growth factors (IGF) play a role in stem cell homeostasis in different species; for example, they induce budding structures in human gastric organoids [30, 31]. Fibroblast growth factors (FGF7 and FGF10) stimulate the division of skin keratinocytes and intestinal epithelial cells and are essential for the expansion of human skin organoids and mouse gastric organoids [5, 32, 33]. We found that IGF-1 and FGF-10 exerted similar positive effects on rumen epithelial organoids, and the absence of IGF-1 and FGF-10 significantly affected organoid formation. The canonical Wnt pathway relies on β-catenin, and a GSK3β inhibitor can prevent the degradation of β-catenin and further activate the Wnt pathway [34]. Transforming growth factor β (TGF-β) is a secreted pleiotropic factor involved in cell proliferation, differentiation, death, and migration, and TGF-β inhibitors can prolong the culture time of intestinal organoids by maintaining the state of undifferentiated stem cells [35, 36]. EGF signal transduction is negatively regulated by the p38 MAPK pathway, with the loss of p38 promoting the proliferation of intestinal epithelial cells; thus, p38 MAPK inhibitors are often used to stimulate the proliferation and long-term maintenance of human intestinal organoids [37, 38]. Rho-associated protein kinase inhibitors can suppress caspase-dependent cell death, and when culturing isolated single stem cells, ROCK inhibitors are usually added to the medium to prevent anoikis [3]. The ROCK inhibitor Y-27632, p38 MAPK inhibitor SB202190, TGF-β inhibitor A83-01, and GSK3β inhibitor CHIR-99021 have been reported to improve the culture of human, porcine, and bovine intestinal organoids [36, 39–41]. During the culture of rumen epithelial organoids, Y-27632, SB202190, A83-01, and CHIR-99021 were found to be essential for organoid initiation and long-term maintenance, and removal of any inhibitor resulted in deterioration of the organoids.

The components of the animal cytoskeleton, such as intermediate filaments (IF), are evolutionarily highly conserved and very similar within species-specific cells [42]. Keratinocytes in the stratum basale of stratified epithelium express keratin 5 (KRT5) and keratin 14 (KRT14) as the main keratin pair assembled into keratin intermediate filaments (KIF). When keratinocytes differentiate and migrate out of the stratum basale, the expression of KRT5 and KRT14 is turned off [43]. Keratinization occurs in the suprabasal layer, forming the cornified envelope, and involucrin (IVL), as the main component of the cornified envelope, is exposed outside the membrane of the cells in the stratum granulosum and stratum corneum [23, 44]. ZO-1 is an integral membrane protein that is expressed in a variety of mammalian stratified epithelia (skin, esophagus, vagina, and rumen), and its expression level gradually decreases from the stratum corneum to the stratum basale [16, 45]. Similar results were observed in our study: the localization of KRT14^+^ keratinocytes, Ki67^+^ proliferative cells, and IVL^+^ differentiated keratinocytes in organoids and the fluorescence intensity changes of ZO-1 along the stratified epithelium demonstrated that rumen epithelial organoids possess tissue-like stratified epithelium with inward polarity. The papillae are a classic morphological feature of the rumen epithelium that expand the surface area for nutrient absorption and metabolism [15]. The formation of rumen papillae relies on the expression of *TBX3* transcription factor by papillary fibroblasts adjacent to the epithelium [22], which may explain the lack of similar structures in organoids cultured in vitro. A recent study reported that artificially sculpted gels can promote in vitro intestinal stem cells patterning along predefined spatial boundaries [46], which provides an idea for cultivation of rumen organoids that are close to in vivo morphology.

We compared the expression of cell-specific genes in organoids and tissue from 5-day-old lambs. The organoids we generate represent the epithelial layer of the origin organ, so expression of non-epithelial cells such as immune cells and fibroblasts is absent in the organoids. *JUN*, *FOS* and *ZFP36* are specifically expressed in keratinocytes and participate in cell differentiation [47, 48]. *ANXA1*, *S100A12*, *CNFN*, and *DSP* are involved in the process of cell keratinization [49–52]. By combining the expression of rumen epithelial cell lineage-specific genes in organoids and tissue, we speculate that keratinocytes undergo a differentiation process from the stratum basale to the stratum corneum in organoids. Expression levels of *ACAT1* and *SLC16A1* associated with ketogenesis, a hallmark of metabolic development in the rumen epithelium [53], were consistent in organoids and tissue. However, there are differences in the expression of *CA1* and *ACAA2*, which are mainly involved in lipid metabolism between organoids and tissue [54], the results of WGCNA also indicate that organoids and tissues have different fatty acid metabolic capabilities, which may be related to the conditions of ex vivo culture. It would be interesting to explore whether optimized culture conditions such as stimulation with specific nutrients and suspension culture can induce the development of lipid metabolic capacity in organoids in future studies.

In the rumen epithelium, tight junctions, gap junctions, adherens junctions, and desmosomes are responsible for intercellular adhesion and aggregation [15, 45, 55], as illustrated by the results of TEM and cell junction-related gene expression in our research. Due to the limitations of ex vivo culture, the structural complexity of organoids is lower than that of tissue, and different extracellular environments may bring different external system forces [56], such as rumen peristalsis and chyme friction, which may lead to a mismatch in the expression levels of some genes related to cell connection between organoids and tissue. At the transition between the stratum granulosum and stratum corneum, the morphology of the desmosome changes, and the cytoplasmic plaques integrate into the cornified envelope to form homogeneous electron-dense plaques in the extracellular core, forming the special desmosomes that are known as corneodesmosomes [57]. Similar results were observed by TEM of the inside of organoids.

The natural architecture of 3D intestinal organoids is that the apical boundaries of the epithelial cells face the central lumen, which limits access to nutrient and microorganisms on the apical surface of the epithelial cells. Polarity reversal of 3D intestinal organoids from chickens, mice, and sheep has recently been reported [10, 58, 59], making in vitro host-pathogen infection models more practical. In addition, 2D organoid cultures derived from 3D organoids can also serve as models for studying epithelial barriers, nutrient absorption and metabolism, and pathogen infection [60, 61]. Rumen epithelial 2D cultures induced from 3D organoids contained KRT14^+^ and IVL^+^ stratified epithelial structures and were able to be maintained for more than 7 days. Tight junctions between epithelial cells were demonstrated by ZO-1 staining. However, more studies are necessary to explore the functionality of 2D cultures, such as permeability and trans epithelial electrical resistance measurements, transcriptome analysis, and the development of protocols for passaging, cryopreservation, and resuscitation of 2D cultures. Extensive investigation of the absorption and metabolism of nutrients and drugs in 2D cultures is a topic for future research.

Because of the reflexive closure of the esophageal groove [62], the rumen of the newborn milk-fed ruminant lacks substrates for decomposition, and all the energy required comes from the glucose, lactose, milk fat and amino acids from the milk replacer absorbed into the blood through the small intestine. Early in vitro experiments demonstrated that glucose is not a favored energy substrate in neonatal rumen epithelial cells [63], suggesting that the rumen epithelium may be more inclined to use fatty acids and amino acids as substrates under the condition of milk replacer feeding, which is consistent with the KEGG enrichment results of MEred. The KEGG enrichment results of MEblue indicate that carbohydrate metabolism is vigorous in organoids. We speculate that this is because the exogenous oxidizable substrate added to the organoid medium is glucose. Rumen epithelial ketogenic capacity gradually increases with age in suckling lambs in the absence of solid feed [64, 65], the lack of enrichment of pathways related to ketone body metabolism in tissue and organoids may be due to the lack of related substrates, such as SCFA. According to the research of Yuan et al. [22], the stratum spinosum with mature metabolic function is established in lambs before weaning, and this process takes approximately 45 days. However, the time of in vitro organoid culture maintenance is limited, which may be the reason for the loss of metabolical function in organoids.

Genes in MEblue exhibit opposite expression levels in tissues and organoids. Among the pathways enriched from MEblue, the FoxO signaling pathway and p53 signaling pathway are related to cell cycle arrest, cellular senescence and apoptosis [66, 67], and the Hippo signaling pathway is involved in the control of cell growth and organ size [68]; thus, these pathways may be involved in growth restriction in the later stage of organoid culture. For this reason, it is worth noting that the AMPK signaling pathway and mTOR signaling pathway related to the regulation of metabolism and maintenance of cell homeostasis may be involved in the maintenance of organoids [69, 70].

In the skin, keratinocytes recruit immune cells and regulate their survival and retention in the tissue. After resolution of infection, resident memory T cells (TRM) form and disperse in the skin to provide protective immunity and are particularly dependent on epithelial-derived factors, such as IL-7 and IL-15, supplied by keratinocytes [71–73]. Keratinocytes are also an important source of cell survival signals in the innate immune network, and colony-stimulating factor 1 (CSF1) receptor signaling and its ligand CSF1 support the development and maintenance of macrophages [74]. The retention of immune cells in the epidermis is dependent on TGF-β signaling, which is activated by keratinocytes through the production of integrins [75, 76]. In the rumen epithelium, spinous cells induce lymphocyte proliferation to generate immune responses, and the maturation of the stratum spinosum is very important for the establishment of immune function [22]. However, organoids induced from adult stem cells stably retain distinct cellular phenotypes from the tissue of origin [77, 78], making the absence of immune cells inevitable in epithelial organoids. Although the intestinal crypts used to grow organoids contain immune cells, they are lost during subsequent cultures [11]. In our study, the expression level of genes in MEyellow increased with age in rumen tissue but was low in organoids, and the enriched KEGG pathways were related to the immune system, indicating that rumen immune function is gradually established with age in lambs, whereas in organoids, immune function is always lost.

Through WGCNA, we found that some eigengenes were expressed in opposite trends between organoids and tissues at day 5, indicating that there are differences in the transcriptional profiles of organoids and tissue, which may be due to more complex cell types in tissues. We predict that the ex vivo sterile culture system may also be responsible for the differences in transcript profiles. In future studies, it will be necessary to verify whether specific nutrients from food or stimulation from microorganisms will promote the development of organoid functions such as immunity and metabolism.

In this work, we demonstrate the feasibility of expanding single rumen epithelial cells into organoids in vitro. We substantiate the fatty acid absorptive capacity of organoids and generated 3D-derived 2D cultures. We validated the necessity of individual components in rumen epithelial organoid culture conditions. We demonstrate that rumen organoids have tissue-like stratified epithelial features by immunofluorescence and transmission electron microscopy. Through RNA-seq, we identified rumen cell type-specific markers in organoids and explored differences between organoids and tissue. In conclusion, we established a novel in vitro model of the rumen epithelium that will help us gain a better view of rumen epithelial cell function while potentially reducing the use of experimental animals.

### Supplementary Information

**Additional file 1:** **Supplementary immunofluorescence staining**. **A** Control staining of cells, organoids, and tissue; rabbit IgG was used in place of the primary antibody followed by Cy3-conjugated secondary antibody and DAPI (nuclear marker) labelling. **B** Immunostaining of Involucrin (IVL) and DAPI for cells. Scale bars = 100 μm.

**Additional file 2:** **2D cultures induced from 3D organoids**. **A** Representative images of 3D-derived 2D cultures on day 7, 14, 21. **B** Representative images of immunofluorescent staining of longitudinal sections of 2D cultures. **C** Fatty acid uptake of 2D cultures. Scale bars = 100 μm.

## Data Availability

The raw sequencing data generated in this study are publicly available in NCBI Sequence Read Archive under the accession number PRJNA941374.
