# Supplementary figures and images for: 3D sheep rumen epithelial structures driven from single cells in vitro
Source: Vet Res. 2023 Nov 9;54:104. doi: 10.1186/s13567-023-01234-1 (PMC10636852; doi:10.1186/s13567-023-01234-1)

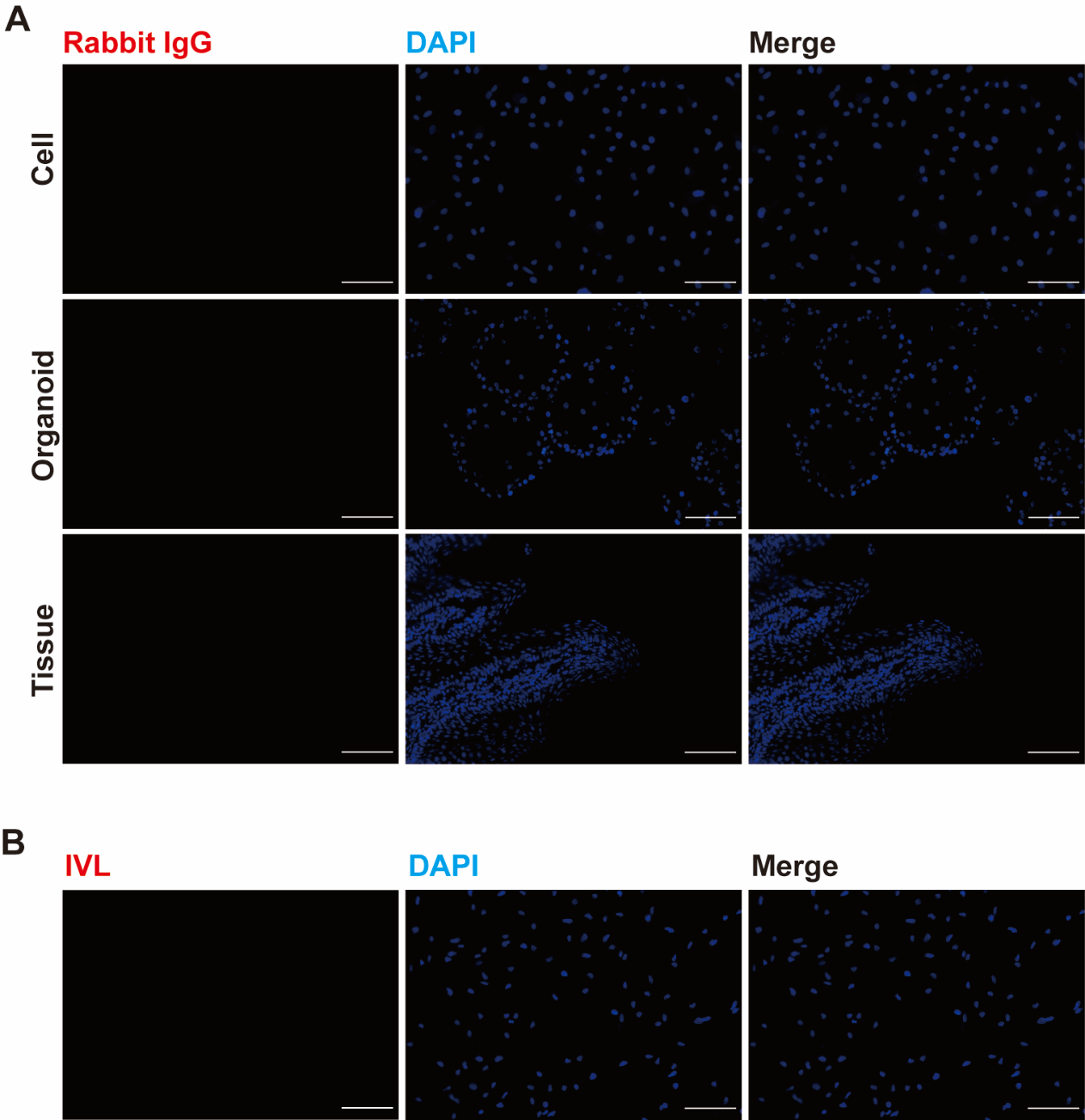

Supplement: Supplementary file 1 — Additional file 1: Supplementary immunofluorescence staining. A Control staining of cells, organoids, and tissue; rabbit IgG was used in place of the primary antibody followed by Cy3-conjugated secondary antibody and DAPI (nuclear marker) labelling. B Immunostaining of Involucrin (IVL) and DAPI for cells. Scale bars = 100 μm. [file 13567_2023_1234_MOESM1_ESM.docx]

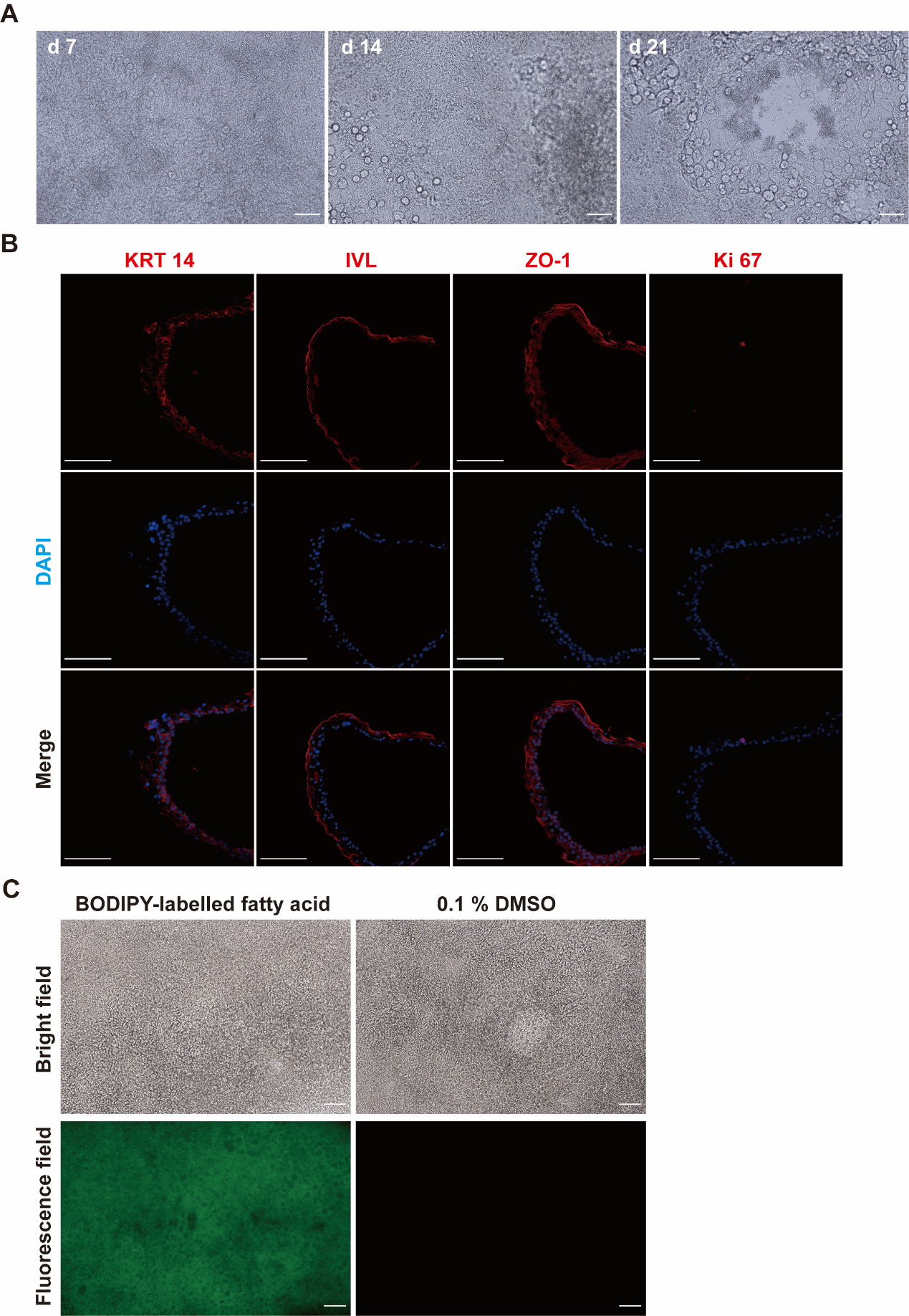

Supplement: Supplementary file 2 — Additional file 2: 2D cultures induced from 3D organoids. A Representative images of 3D-derived 2D cultures on day 7, 14, 21. B Representative images of immunofluorescent staining of longitudinal sections of 2D cultures. C Fatty acid uptake of 2D cultures. Scale bars = 100 μm. [file 13567_2023_1234_MOESM2_ESM.docx]
